# Comparative effectiveness of group-analysis therapy and psychoeducation in patients with different somatoform disorders

**DOI:** 10.1192/j.eurpsy.2022.493

**Published:** 2022-09-01

**Authors:** A. Tkhostov, E. Rasskazova, I. Belokrylov

**Affiliations:** 1 Moscow State University, Clinical Psychology, Mokhovaja, Russian Federation; 2 Mental Health Research Center, Medical Psychology, Moscow, Russian Federation; 3 Moscow State University, Clinical Psychology, Moscow, Russian Federation; 4 Medical Institute of the People’s Friendship University of Russia, Department Of Psychiatry And Medical Psychology, Moscow, Russian Federation

**Keywords:** group analysis, illness represenation, Somatoform disorders

## Abstract

**Introduction:**

Psychological interventions including group analysis (Leichsenring et al., 2015, Beutel et al., 2008) are effective with patients having somatoform disorders.

**Objectives:**

To reveal differences in dynamics of pathological bodily sensations, quality of life, illness representation in patients with somatoform disorders undergoing group analysis and psychoeducation program.

**Methods:**

100 patients with somatoform disorders (undifferentiated somatoform disorder – 42, somatization disorder – 10, somatoform autonomic disfunction – 36, persistent somatoform pain disorder and other SD – 12) were randomly assigned randomized to psychoeducation intervention and to the group analysis psychotherapy. Before and after treatment they filled Screening for somatoforms symptoms (Rief, Hiller, 2003), Illness Perception Questionnaire - Revised (Moss-Morris et al., 2002), Cognitions About Body And Health Questionnaire (Rief et al., 1998), Scale for the Assessment of Illness Behaviour (Rief et al., 2003), Quality of Life Enjoyment and Satisfaction Questionnairie-18 (Ritsner et al., 2005).

**Results:**

In both conditions decrease in complaints was the most in patients with undifferentiated somatoform disorder and the least in somatoform autonomic disfunction (F=6.19, p<.01, η²=.17). In patients with somatization disorder there was the most increase in quality of life in leisure time, beliefs about intolerance to bodily sensations, rechecking the diagnosis (F=3.32-4.87, p<.05, η²=.10-.14). Decrease in beliefs about bodily weakness, illness consequences was the most prominent in patients with somatization disorder undergoing group therapy (F=2.90-4.46, p<.05, η²=.09-.13).

**Conclusions:**

Patients with undifferentiated somatoform disorder demonstrate most clinical improvement in interventions while patients with somatization disorder – the most psychological improvement. Research is supported by the Russian Foundation for Basic Research, project No. 20-013-00799.

**Disclosure:**

Research is supported by the Russian Foundation for Basic Research, project No. 20-013-00799.

